# The Potential Role of Glutamate Receptors and their Antagonists or Modulators in Migraine Therapy

**DOI:** 10.2174/1570159X23666250403124115

**Published:** 2025-04-04

**Authors:** Chih-Hung Tsai, Ming-Chi Lai, Chin-Wei Huang

**Affiliations:** 1 Department of Neurology, National Taiwan University Hospital Yunlin Branch, Yunlin, Taiwan;; 2 Department of Pediatrics, Chi-Mei Medical Center, Tainan, Taiwan;; 3 Department of Neurology, National Cheng Kung Univesity Hospital, College of Medicine, National Chen Kung University, Tainan, Taiwan

**Keywords:** Migraine, inotropic glutamate receptors, metabotropic glutamate receptors, glutamate receptor antagonists, glutamate receptor modulators, cortical spreading depression

## Abstract

**Background:**

Glutamate is implicated in playing a crucial role in modulating the complex pathophysiological mechanisms of migraines, including central or peripheral sensitization, cortical spreading depression, and pain transmission. With expanding knowledge over the last three decades, glutamate receptors have become focal points in neurological drug research. Altered plasma glutamate levels during migraines suggest a potential avenue for effective therapies targeting glutamate reduction. Furthermore, glutamate is believed to play a vital role in modulating the complex pathophysiological mechanisms underlying migraines.

**Objective:**

This study aims to provide an overview of the ionotropic glutamate receptor antagonists (NMDA, AMPA, and Kainate receptors) and metabotropic glutamate receptors in the context of migraines. We explore the advantages and disadvantages of these receptor modulators as alternative treatments, considering efficacy, tolerability, and safety.

**Methods:**

We conducted comprehensive online searches across various electronic databases, with a primary focus on PubMed and clinicaltrials.gov, to gather the latest treatment approaches and emerging concepts.

**Results:**

A total of 371 articles were identified from PubMed, along with 69 articles from clinicaltrials.gov. After refinement, 113 articles were included. We summarize seven different medications currently in clinical practice for migraines and highlight six items for migraine therapy in preclinical trials and their potential value.

**Conclusion:**

It's crucial to note that these agents pose certain challenges in specific drug research due to their intricate influence and mechanisms of action within multiple neuronal pathways. Therefore, further studies are warranted to elucidate more specific glutamatergic signaling pathways for migraine therapy while minimizing interference with normal neuronal functions.

## 1 INTRODUCTION

Migraine, recognized as a primary headache disorder, manifests as recurrent, moderate to severe headaches localized to one side of the head. These headaches are often accompanied by specific autonomic dysfunctions, such as nausea, vomiting, photophobia, and phonophobia. Migraine is more prevalent among females and imposes a substantial socio-economic burden on the healthcare system [1].

The precise mechanisms underlying migraine attacks remain elusive. Currently, the pathogenesis of migraine is mostly attributed to trigeminovascular activation and heightened excitability in various regions of the Central Nervous System (CNS) involving neurotransmitters [2-4]. Neurotransmitters like glutamate, Calcitonin Gene-Related Peptide (CGRP), serotonin, γ-aminobutyric acid (GABA), substance P, and Neurokinin A play pivotal roles in both migraine attacks and migraines with aura, primarily triggered by cortical spreading depression (CSD) [5, 6]. In clinical practice, migraine treatment is generally divided into two main categories: acute relief and prophylactic therapy [7]. Initially, painkillers such as Acetaminophen and non-steroidal anti-inflammatory drugs are typically recommended for acute management. In cases of insufficient relief or severe attacks, triptans (5-HT1-receptor agonists) are considered an alternative abortive option. Prophylactic agents are employed to reduce the frequency and intensity of migraine attacks, especially when they occur more than twice a month or when adverse effects persist despite first-line treatments. These prophylactic agents may include beta-blockers (*e.g*., Propranolol), calcium channel blockers (*e.g*., Flunarizine), antidepressants (*e.g*., Amitriptyline), and anti-epileptic drugs (*e.g*., Valproic acid/Topiramate) [8-10]. Additionally, CGRP antagonists and Onabotulinum Toxin A are selected for treating chronic migraine [7, 11-13]. Nevertheless, there remain limitations associated with these various treatment options, such as intolerable side effects and limited efficacy. Recent reviews have indicated elevated serum glutamate levels during migraine attacks, both in the interictal and ictal periods. Glutamate is a widely distributed excitatory neurotransmitter within the CNS [14, 15]. Moreover, effective migraine therapies have been shown to reduce plasma glutamate levels. Hence, glutamate is recognized as a unique and potentially significant factor in modulating the pathophysiology of migraine. In this article, we will delve into the potential role of glutamate receptors, their antagonists, and modulators in both preclinical and clinical migraine therapies.

## 2 MATERIALS AND METHODS

We conducted comprehensive online searches across various electronic databases, with a primary focus on PubMed and clinicaltrials.gov, in order to gather the latest treatment approaches and emerging concepts. Our search encompassed abstracts, randomized controlled trials, and reviews up to April 2023. To ensure a thorough investigation, we employed a range of keywords, both independently and in combination, including the terms “Migraine”, “therapy”, “glutamate receptors”, “modulators”, “NMDA”, “AMPA”, “kainate receptor”, and “kynurenine”. Additionally, we refined our search results using logical operators such as “not”, “and”, and “or”. Our selection criteria dictated that all chosen articles must be in the English language.

In the initial phase of our selection process, we identified a total of 371 articles from PubMed and 69 articles from clinicaltrials.gov. Subsequently, we excluded 240 articles that were not written in English or did not align with our specified keywords related to glutamate and migraine.

**Fig. (1):**
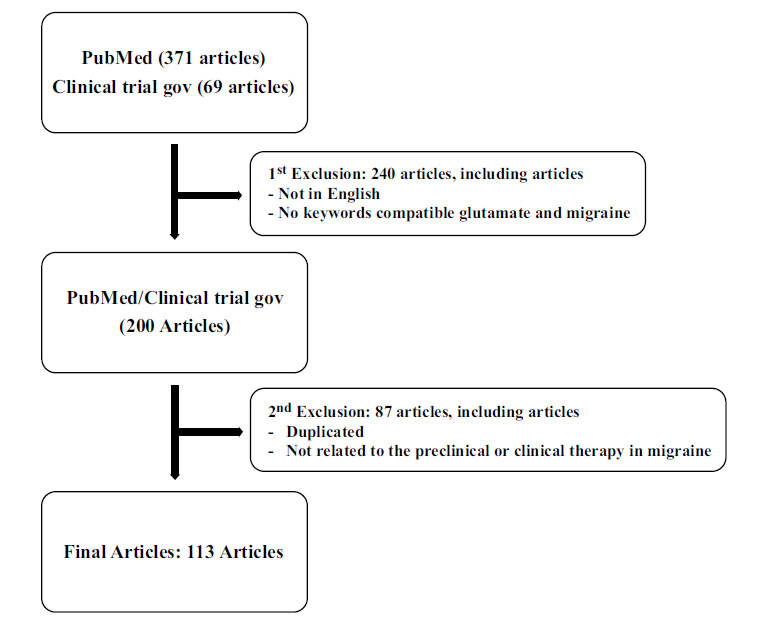
Research flowchart.

In the subsequent stage of our selection criteria, we further refined our selection to include 113 articles for inclusion in this review. We excluded 87 articles due to reasons such as duplicate data and lack of relevance to either preclinical or clinical migraine therapy (Fig. **1**).

## 3 RESULTS AND DISCUSSION

### 3.1 The Potential Role of Glutamate in Migraine

In the CNS, both glutamate and GABA play pivotal roles as neurotransmitters, serving as excitatory and inhibitory agents, respectively. Within the cerebral cortex, the predominant neuronal population relies on glutamate for its functioning [16]. These numerous glutamate receptors are broadly categorized into two major groups: ionotropic glutamate receptors (iGluRs) and metabotropic glutamate receptors (mGluRs), each employing distinct modes of modulation [17]. Ionotropic glutamate receptors function as ligand-gated ion channels and can be further classified into three major families: N-Methyl-D-Aspartate (NMDA), α-amino-3-hydroxy-5-methylisoxazole-4-propionate (AMPA), and 2-carboxy-3-carboxymethyl-4-isopropenylpyrrolidine (kainate/ KA) receptors [18, 19]. On the other hand, metabotropic glutamate receptors (mGluRs) belong to the G protein-coupled receptors superfamily (mGlu1~mGlu8) [20, 21].

**Fig. (2):**
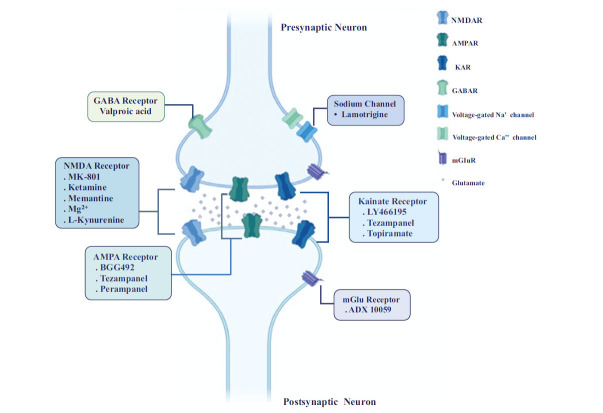
Different glutamate receptors are widely located in neurons, including pre- or post- synaptic areas. Preclinical/ clinical glutamate receptor antagonists or modulators and their target sites are illustrated.

Glutamate is believed to play a multifaceted role in the pathophysiology of migraine. Among its various functions, CSD stands out as a significant contributor to migraine with aura. CSD initiates neuronal depolarization, which subsequently spreads throughout the cortex [6]. Additionally, glutamate is implicated in central sensitization in the thalamus and peripheral sensitization in the somas of trigeminal ganglion neurons or meningeal trigeminal nerve terminals [22-24]. Furthermore, during an episodic migraine attack, plasma glutamate levels rise, while cerebrospinal fluid (CSF) levels of glutamate increase in cases of chronic migraine during the interictal period [14, 15]. These observations suggest a potential correlation between elevated glutamate levels and trigeminal-neuronal activation [25]. Moreover, effective prophylactic migraine treatments have been shown to reduce serum glutamate levels in certain preclinical studies. Additionally, some studies have indicated that consuming Monosodium Glutamate (MSG) may trigger migraines due to increased glutamate levels [26, 27]. Furthermore, there is evidence implicating central sensitization, in which the mGlu5 receptor plays a role by modulating synaptic transmission in the trigemiocervical nucleus. Ionotropic glutamate receptors primarily regulate fast synaptic transmission, whereas mGluRs mediate intracellular second messenger systems and trigger postsynaptic excitability, leading to glutamate release [28]. Subsequent preclinical and clinical investigations into glutamate receptor antagonists and modulators have revealed their potential anti-migraine effects (Table **1** and Fig. **2**).

### 3.2 NMDA Receptors

NMDA receptors, a prominent subgroup of ionotropic glutamate receptors, are widely distributed throughout the CNS, with a notable concentration in the superficial laminae of the Trigeminal Nucleus Caudalis (TNC) [29]. These receptors are primarily located in the postsynaptic membrane, although a few have been observed in presynaptic and extrasynaptic membranes [30]. NMDA receptors are known to modulate various excitatory neuronal responses within the CNS and play a crucial role in essential neuronal functions such as cognition, movement, sensory processing, and emotional regulation [31, 32]. Furthermore, NMDA receptors have been associated with specific neuropsychiatric conditions, including epilepsy, stroke, mild cognitive impairment, dementia, schizophrenia, Parkinson's disease, addiction, pain, and migraine [33].

A key aspect of NMDA receptor activation is the influx of calcium ions (Ca^2+^). Typically, magnesium ions (Mg^2+^) block the channel pore to prevent the influx of cations, particularly Ca^2+^ and are considered selective inhibitors of glutamate-induced cortical spreading depression. When Mg^2+^ leaves its original binding site, excessive Ca^2+^ can flow through the pore, leading to neuronal depolarization [34]. However, prolonged and excessive Ca^2+^ influx into neurons can result in cellular damage or even cell death. NMDA receptors are modulated by glutamate in conjunction with co-agonists such as glycine or D-serine [35]. Additionally, NMDA receptors can be categorized into three genetic groups: GluN1, GluN2A-D, and GluN3A-B. Among these subgroups, GluN2B NMDA receptors, primarily found in nerve fibers, play a crucial role in innervating dural blood vessels, signaling pain transmission, and exhibiting higher glutamate levels during migraine attacks, which are reduced after migraine therapy [36, 37]. As CSD progresses, some preclinical studies have indicated that presynaptic NMDA receptors are activated, leading to increased glutamate release [38].

Moreover, two distinct types of NMDA receptor antagonists have shown promise in migraine management. The first type includes non-competitive channel blockers like MK801 and ketamine, which have been effective in halting the propagation of CSD. The second type consists of competitive glutamate antagonists, such as DL-2-amino-7-phosphonoheptanoate, which can inhibit the spread of depression in parieto-occipital areas [39]. In essence, there appears to be a correlation between CSD and NMDA receptor activation, suggesting that NMDA receptor antagonists may hold potential as a therapy for migraine with aura.

Furthermore, recent animal studies conducted using an inflammatory soup method involving the local injection of glutamate to induce focal inflammation and cause paresthesia have suggested a link between NMDA receptors and peripheral sensitization. These studies demonstrated that NMDA receptor antagonists, including MK-801, were able to alleviate paresthesia [40].

### 3.3 AMPA Receptors

In comparison to NMDA receptors, AMPA receptors are relatively less abundant in the CNS. AMPA receptors are primarily situated in the postsynaptic membrane, especially within the superficial laminae of the TNC [41]. They play a pivotal role in facilitating fast excitatory synaptic transmission by binding to glutamate in the cell membrane. AMPA receptors are composed of heterotetramers containing four subunits: GluA1, GluA2, GluA3, and GluA4 [42]. Neuropathic pain and central sensitization are triggered by the dynamic trafficking of GluA1-containing AMPA receptors rather than those containing GluA2/3/4, thereby exerting control over synaptic plasticity [43]. Furthermore, the activation of GluA1-containing AMPA receptors is believed to be associated with chronic migraine. Among these subunits, GluA2 is considered the primary receptor responsible for modulating ligand-gated ion channels [44].

There are two distinct types of AMPA receptors, depending on the presence of the GluA2 subunit in the tetramer complex, which in turn influences Ca^2+^ activity at the cell membrane [42, 45]. Typically, the predominant type is the GluA2-containing AMPA receptors, which are Ca^2+^-impermeable (CI) and are predominantly found in the CNS [42, 46]. These CI AMPA receptors play a crucial inhibitory role by preventing the influx of Ca^2+^ and Zn^2+^. In contrast, GluA2-lacking AMPA receptors are often Ca^2+^ permeable (CP) and are relatively atypical, being less commonly observed in the mature brain.

Recent preclinical and clinical investigations have demonstrated that the AMPA receptor antagonist GYKI52466 can inhibit nociceptive signaling in the TCC (trigeminal cervical complex). Additionally, another antagonist targeting AMPA/ kainite receptors, LY293558, has been shown to reduce the expression of c-fos in the TCC. Both of these compounds appear to be effective in controlling migraine attacks [47, 48]. Consequently, current research suggests a potential pharmacological mechanism involving AMPA receptors in the context of migraine.

### 3.4 Kainate Receptors

Kainate (KA) receptors, belonging to the non-NMDA group of receptors, are distributed widely in both pre-and post-synaptic regions that are involved in central nociceptive signaling within the trigeminal ganglion. They differ from NMDA or AMPA receptors, which are primarily found in post-synaptic regions [49-51]. Due to the lack of specific pharmacological interventions, our understanding of the detailed functions and mechanisms of KA receptors remains limited compared to NMDA and AMPA receptors.

KA receptors are divided into five subunits (GluK1-5), each displaying two distinct affinities and potencies for neuronal activation and desensitization [49]. The first group comprises low-affinity subunits, including GluK1-3, which can assemble as functionally homomeric or heteromeric ion channels to modulate kainate. On the other hand, high-affinity subunits such as GluK4 and GluK5 can form pragmatic heteromeric channels for kainate assembly in the cell membrane [49].

Presynaptic KA receptors have a role in mediating both bi-directional GABA and glutamate release, influencing nociceptive neurotransmission and pain modulation. Postsynaptic KA receptors play a crucial role in mediating small currents with slow kinetic activation, contributing to synaptic transmission [49-51]. In animal models of peripheral pain, the activation of KA receptors is associated with thermal and mechanical paresthesia [52, 53]. Preclinical studies have suggested that GluK1 receptor antagonists are more effective in eliciting antinociceptive responses compared to selective AMPA antagonists [49]. Additionally, LY466195, a selective GluK1 antagonist, has shown potential as an anti-migraine agent by inhibiting nociceptive neuronal activity in the TCC, which in turn leads to neurogenic dural vasodilation [49, 54].

### 3.5 Metabotropic Glutamate Receptors

Our understanding of the role of mGluRs in the pathophysiology of migraine remains incomplete. Generally, mGluRs can be classified into different groups based on their subtypes and distribution patterns. Group I mGluRs, consisting of mGluR1 and mGluR5, are primarily located postsynaptically and play a role in regulating phospholipase C activity, which leads to increased neuronal excitatory transmission and enhanced glutamate release [18]. In contrast, Group II mGluRs (mGluR2 and mGluR3) and Group III mGluRs (mGluR4/mGluR7 and mGluR8) are predominantly situated presynaptically. They contribute to antinociceptive responses by activating adenylyl cyclase, ultimately reducing glutamate release. In the context of migraine, mGluR5 is believed to be associated with inflammation and the chronicization of neuropathic pain [55, 56]. A clinical trial involving ADX 10059, a negative allosteric modulator of mGluR5, demonstrated decreased dural vasodilation and reduced nociceptive neurotransmission through the TCC [57] (Table **2**).

As for Group II and III mGluRs, these receptors not only decrease glutamate release but also modulate GABA inhibition, leading to neuronal disinhibition. However, current preclinical studies involving Group II and III mGluRs face challenges due to the lack of more specific antagonists or agonists that would allow for a more detailed evaluation of each isolated mGluR function and the complex mechanisms involved [2, 58].

### 3.6 NMDA Receptor Antagonists

#### 3.6.1 MK-801

MK-801 (Dizocilpine maleate) is a preclinical trial antagonist of NMDA receptors. It has been suggested that MK-801 can suppress the initiation and propagation of CSD to some extent, making it potentially effective in managing migraine with aura [39, 59].

In animal studies focused on central sensitization, intravenous administration of MK-801 has demonstrated the ability to reduce Fos protein immunoreactivity in the TCC. This reduction is observed in response to a chemical reaction triggered by mustard oil, which is related to corneal nociceptors. This suggests that NMDA receptor antagonists may play a significant role in modulating the transmission of trigeminal nociceptive signals [59-62].

Furthermore, in animal models involving peripheral stimulation, pretreatment with MK-801 has been shown to alleviate peripheral sensitization, such as allodynia or paresthesia induced by local injections of glutamate or carrageenan [53]. Both central and peripheral sensitization processes are closely linked to the chronification of migraine, and the preclinical trials with MK-801 indicate its potential efficacy in migraine management [61, 62].

### 3.7 Endogenous NMDA Receptor Antagonist

#### 3.7.1 L-Kynurenine (LKYN)

The fundamental metabolism of tryptophan primarily relies on the kynurenine pathway (KP), which gives rise to multiple metabolites, including L-kynurenine (LKYN), kynurenic acid (KYNA), quinolinic acid, xanthurenic acid, and nicotinamide [63]. The KP has been suggested to play a crucial role in various neuronal mechanisms and CNS phenomena, such as epilepsy, stroke, dementia, and migraine [64, 65]. Among these metabolites, LKYN is the initial product and serves as the precursor for other kynurenine metabolites. However, KYNA is a downstream metabolite derived from LKYN and stands out as the only endogenous NMDA receptor antagonist [66, 67]. Nevertheless, KYNA has a natural limitation as a neuroprotective agent due to its poor permeability through the blood-brain barrier (BBB) [68]. In contrast, LKYN can easily cross the BBB under physiological conditions. Moreover, the administration of LKYN *via* a peripheral route can raise the intracranial concentration of KYNA, thereby modulating the function of NMDA receptors. This potential approach may lead to therapies for various unclear neurodegenerative disorders, epilepsy, migraine, and stroke [69, 70].

In a Phase I study conducted on healthy volunteers in 2021 [66], intravenous infusion of LKYN demonstrated safety and good tolerability up to a dosage of 5 mg/kg. It is considered a potential therapeutic agent for epilepsy and migraine, although further extensive studies are needed to elucidate the detailed and unclear mechanisms within the human brain.

### 3.8 AMPA Receptor Antagonists

#### 3.8.1 BGG492

In a prior randomized, double-blind trial aimed at assessing the acute treatment of migraine [3], BGG492, a specific AMPA receptor antagonist administered orally at a dose of 250 mg, exhibited no significant differences in primary and secondary outcomes when compared to sumatriptan (administered subcutaneously at a 6 mg dosage) and placebo. However, it's worth noting that the rates of adverse events were higher in the BGG492 group than in the sumatriptan and placebo groups, with reports of mild to moderate dizziness, vertigo, and gait disturbance being more frequent [71]. In other words, it appears that isolated AMPA receptor antagonists like BGG492 may have limited efficacy in migraine therapy, possibly due to their lack of impact on the peripheral trigemino-vascular system [72, 73].

### 3.9 Kainate Receptor Antagonists

#### 3.9.1 LY466195

The preclinical kainate receptor antagonist, LY466195, has been considered a potential therapy for the acute treatment of migraine [3, 49, 74]. However, when compared to placebo and sumatriptan (administered in a 6mg subcutaneous form), LY466195 (given intravenously at doses of 1mg or 3mg) did not meet the primary endpoints, particularly with regard to the response rate at 2 hours. Furthermore, LY466195 only demonstrated a higher secondary efficacy (pain-free rate of 29% compared to 0% with placebo) but was less effective than sumatriptan (29% *vs*. 50%) [3]. Notably, LY466195 not only exhibited reduced efficacy for acute migraine relief but also resulted in side effects, including visual disturbances, as observed in the trial [3, 49, 74, 75, 76]. These findings may suggest that exploring the potential role of kainate receptors as novel targets for investigating acute abortive agents is an avenue worth pursuing.

#### 3.9.2 Tezampanel (NGX424/LY293558)

Tezampanel (NGX424/LY293558), a clinical trial medication belonging to the class of competitive AMPA/GluK1 receptor antagonists, has not yet received approval from the US Food and Drug Administration (FDA) (currently in Phase II for migraine) [3, 49]. Previous studies have demonstratedthat intravenous administration of tezampanel at a dose of 1.2 mg/kg result in better primary efficacy outcomes (response rate at 2 hours: 69% *vs.* 25%) and secondary efficacy outcomes (pain-free rate at 2 hours: 54% *vs.* 6%) when compared to placebo. However, it did not outperform oral sumatriptan (administered at 6 mg) in the acute treatment of migraine (1^st^ efficacy comparison: Tezampanel *vs*. sumatriptan - 69% *vs.* 86%; 2^nd^ efficacy comparison: Tezampanel *vs*. sumatriptan - 54% *vs*. 60%) [3]. Additionally, common and bothersome adverse effects have been observed following the use of tezampanel, including dizziness and drowsiness [3, 77].

### 3.10 mGlu5 Receptor Modulators

#### 3.10.1 ADX10059

ADX10059, a negative allosteric modulator, is regarded as a potential therapeutic agent with applications in various fields such as pain management, epilepsy, and neurodegenerative disorders. In a randomized, double-blind trial conducted for migraine, ADX10059 demonstrated superior primary outcomes compared to placebo, particularly in achieving pain-free responses at 2 hours (ADX10059 *vs*. placebo: 16% *vs*. 0%). However, the use of ADX10059 was associated with significant adverse effects and a higher incidence rate, including visual disturbances, dizziness, and vertigo. Ultimately, ADX10059 failed to progress beyond phase II trials due to concerns related to liver toxicity [3, 57, 78, 79].

### 3.11 Prophylactic Therapy for Migraine

#### 3.11.1 Valproic Acid

Valproic Acid (VPA) is a widely used medication employed in the treatment of epilepsy, bipolar disorders, and migraine [7]. As an anti-seizure medication (ASM), VPA primarily acts through the GABA system to inhibit excitability within the cortical neuronal network. Recent studies have revealed that glutamate-induced excitotoxicity can lead to neuronal damage and may play a pivotal role in various neurodegenerative disorders.

In a particular preclinical study [80], it is suggested that VPA may possess antioxidant properties, which could result in a reduction in glutamate-induced excitotoxicity. This effect is indicated by experimental observations of decreased oxidative parameters, including hydrogen peroxide and malondialdehyde (MDA). However, the impact on superoxide dismutase (SOD) and cell catalase (CAT) in SH-SY5Y cells was found to be less pronounced [81-83]. Therefore, in the context of migraine prevention, VPA not only increases the activation of GABA to suppress neuronal transmission but also has the potential to inhibit glutamate-induced excitotoxicity.

#### 3.11.2 Topiramate

Topiramate is commonly prescribed as an ASM, but its pharmacological mechanism remains somewhat unclear due to its complex neuronal modulation. This includes the elicitation of GABA excitation, inhibition of L-type calcium and voltage-gated sodium/potassium channels, and the blocking of AMPA/KA receptor activation [49, 84-87]. Overall, topiramate is considered to have potential neuroprotective and anti-seizure effects.

Interestingly, some clinical reviews have suggested that topiramate is also effective in preventing migraines [2, 3, 84]. Moreover, topiramate plays a crucial role in inhibiting cortical spreading depression and mitigating nitroglycerin-induced hyperalgesia in migraine models. Furthermore, it has been found to effectively control Kainate-induced seizures, particularly in response to GluK1 subunits, although it may not have the same impact on NMDA or AMPA-related seizures. This indicates its potential as a specific and selective treatment option for partial or generalized tonic-clonic seizures, as well as migraine [88, 89]. Additionally, topiramate partially modulates the antinociceptive effect on KA receptors in the TCC [2, 3, 49, 89].

#### 3.11.3 Lamotrigine

Lamotrigine is recognized as an antiepileptic drug that operates by modulating voltage-sensitive sodium channels to inhibit glutamate activation [90]. Regarding its role in prophylactic migraine therapy, there are still some controversial findings. Nevertheless, lamotrigine has demonstrated its effectiveness in reducing the frequency and duration of migraines with aura [91-93]. It is important to note, however, that the use of lamotrigine should be approached cautiously due to potential adverse effects, particularly the risk of skin rash or Stevens-Johnson syndrome (SJS) [3, 93].

#### 3.11.4 Ketamine

Ketamine, a non-competitive NMDA receptor antagonist, modulates the activation of multiple neurotransmitters [94]. It is generally believed to be involved in various pathophysiological conditions, including its anti-epileptic effects, analgesic properties, impact on psychiatric presentations, and amnesic effects. Some research also suggests that ketamine can influence the activation of other receptors, such as opioid, nicotinic, and muscarinic receptors [94, 95].

While studies on ketamine's use as an abortive therapy for episodic migraine have yielded inconsistent results [96, 97], it is considered a potentially effective treatment for refractory chronic migraine or migraine with aura. In clinical practice, ketamine is typically prescribed at initial dosages ranging from 0.1 mg/kg/h to a maximum dosage of 1 mg/kg/h for refractory migraine. However, it is essential to be cautious of potential adverse effects. Furthermore, ketamine plays a crucial role in the antinociceptive function. It holds promise as a treatment option for migraine due to its inhibitory effects on CGRP activation in the TCC and its ability to block cortical spreading depression, as observed in preclinical studies [2, 98].

#### 3.11.5 Memantine

Memantine is primarily known as a major therapy for Alzheimer's disease. It has been reported to modulate neuronal activation *via* NMDA and 5HT3 receptors in previous studies [2, 3, 99]. Therefore, as a member of the NMDA receptor antagonists, memantine has been considered as a potential option for migraine therapy [100]. However, memantine has not yet gained recognition as a preventive therapy for migraine in clinical practice. This may be attributed to its classification as an activity-dependent blocker of NMDA receptors, selectively inhibiting hyperneuronal activation without affecting normal neuronal function [100, 101]. Only a limited number of randomized, placebo-controlled, double-blind studies, such as one involving a daily dose of 10 mg memantine [102], have found it to be relatively effective and safe for patients with migraine. Nonetheless, it is essential to be aware of potential adverse effects associated with memantine use, which may include somnolence, anxiety/depression, increased body weight, and nausea [103]. As of now, memantine has not received approval from the FDA nor is it commonly used in clinical practice for migraine prevention [104, 105].

#### 3.11.6 Magnesium

Several reviews [2, 3, 106, 107] have highlighted the role of magnesium ions in modulating NMDA receptors by primarily blocking excessive calcium influx and inhibiting the propagation of CSD. Conversely, hypomagnesemia can promote excitotoxicity in NMDA receptors, ultimately leading to oxidative stress in the TCC and triggering migraines [108].

In clinical practice, hypomagnesemia has been observed in some migraine cases, and magnesium supplementation has been shown to reduce the frequency of migraine attacks. This is particularly relevant for specific groups of individuals who may be concerned about the adverse effects of typical anti-migraine medications, such as pregnant females, menstrual migraine patients, and elderly individuals with multiple comorbidities [106, 107, 109]. However, it's worth noting that the level of serum magnesium may not accurately reflect the true concentration of intracellular magnesium [108].

While some research has confirmed the pharmacological efficacy of magnesium supplementation and its potential to enhance the effects of existing anti-migraine agents, there remains a lack of detailed guidelines regarding dosage, duration, and the most effective formulations. More extensive studies are warranted to provide further insights into this treatment approach [104, 107].

### 3.12 Acute Abortive Therapy

#### 3.12.1 Perampanel

Perampanel is the first non-selective and non-competitive AMPA receptor antagonist approved by the FDA for the treatment of partial onset seizures, either as monotherapy or adjunctive therapy [110]. A systematic review conducted in 2021 included 31 adult patients with epilepsy and comorbid migraine who were undergoing perampanel treatment. After a 12-month follow-up period, a significant improvement was observed in both epileptic seizures and migraine attacks among the 27 patients who were taking perampanel [111]. These results suggest that perampanel is effective in relieving both epileptic seizures and comorbid migraines when used as a therapy [112].

Some patients reported experiencing psychiatric symptoms such as irritability and aggression while taking perampanel [113]. However, further research is needed to determine the isolated effectiveness and efficacy of perampanel as a treatment for migraines, particularly in larger sample populations and outside the context of epilepsy comorbid with migraines.

## 4 DISCUSSION

According to preclinical studies and clinical research, the pursuit of a safe and viable abortive therapy for migraines through glutamate receptor antagonists or modulators has encountered challenges. While certain clinical glutamate receptor antagonists, such as VPA, topiramate, or magnesium, have been utilized as prophylactic treatments for migraines, recent preclinical and clinical studies reveal a continued lack of more precisely effective glutamate modulators or receptor antagonists for migraine prophylaxis.

This review delves into the potential of mGluR5 receptor modulators and AMPA/kainate receptor antagonists as alternative therapies for migraines, as indicated in recent clinical studies [2, 3, 18, 57]. Additionally, specific NMDA receptor antagonists may exhibit partial efficacy for migraines with aura [93]. Furthermore, kainate receptor antagonists emerge as a promising focus for future investigations, whether in acute abortive measures or preventive treatments for migraines. Kynurenines, newly identified metabolites regulating the endogenous glutamate mechanism, hold potential value for advancing migraine therapy [66].

While various glutamate receptor antagonists or modulators show promise for further exploration and clinical trials in acute abortive or prophylactic migraine treatment, their successful prescription in real-world practice remains elusive due to unexpected adverse effects and non-specific glutamatergic signaling translation.

## CONCLUSION

Based on various preclinical and clinical research findings, there is substantial indirect evidence supporting the notion that glutamate plays a crucial role in modulating the complex pathophysiological mechanisms of migraines, with potential pharmacological implications. It is important to note that glutamate plays essential roles in the normal functioning of the CNS. Currently, there are no specific compounds available that can selectively target glutamate signaling to treat migraine without causing unacceptable adverse effects. Therefore, there is need for further research (*e.g*. L-kynurenine, phase I trial or Perampanel/AMPA receptor antagonist) to explore how to develop more specific glutamatergic signaling interventions for migraine therapy without disrupting normal neuronal function.

## Figures and Tables

**Table 1:** Glutamate receptors/modulators in clinical practice for migraine.

**Drugs**	**Receptor Subtype**	**Function**	**Worse Adverse Effects**
Valproic Acid	GABA/Glutamate Modulator	Prophylactic	Infertility
Topiramate	KA	Prophylactic	Paresthesia; Inattention; Nausea; Fatigue
-	Na Channel/Glutamate Modulator	Migraine with aura	Rash/SJS
Ketamine	NMDA	Chronic migraine Aura	Ataxia; Unreality
Memantine	NMDA	Prophylactic	Depression; Anxiety
Mg^2+^	NMDA Modulator	Alternative	Nil
Perampanel	AMPA	Abortive	Psychiatric symptoms

**Abbreviations**: GABA: γ-aminobutyric acid; KA: kainic acid; NMDA: N-methyl-D-aspartate; AMPA: α-amino-3-hydroxy-5-methylisoxazole-4-propionate; SJS: Stevens-Johnson syndrome.

**Table 2:** Clinical trial as glutamate signaling modulators for acute migraine.

**Study Agent**	**Receptor Subtype**	**Function**	**Comment**
ADX10059	mGluR5	Abortive	Liver toxicity (failure)
Tezampanel	AMPA/GluK1	Abortive	Phase II trial; no FDA approval
LY466195	KA	Abortive	Less efficacy (*vs*. Sumatriptan)
BGG492	AMPA	Abortive	Less efficacy (*vs*. Sumatriptan)
L-kynurenine	NMDA Modulator	Abortive	Phase I trial
MK-801	NMDA	Prophylactic Aura	Role in CSD

**Abbreviations**: KA: kainic acid; NMDA: N-methyl-D-aspartate; AMPA: α-amino-3-hydroxy-5-methylisoxazole-4-propionate; SJS: Stevens-Johnson syndrome; CSD: cortical spreading depression.

## LIST OF ABBREVIATIONS

AMPA: α-amino-3-hydroxy-5-methylisoxazole-4-propionate
ASM: Antiseizure Medication
CAT: Cell Catalase
CGRP: Calcitonin Gene-related Peptide
CNS: Central Nervous System
CSD: Cortical Spreading Depression
CSF: Cerebrospinal Fluid
FDA: US Food and Drug Administration
GABA: γ-aminobutyric acid
KA: Kainic Acid
KP: Kynurenine Pathway
KYNA: Kynurenic Acid
LKYN: L-Kynurenine
MDA: Malondialdehyde
MSG: Monosodium Glutamate
NMDA: N-methyl-D-aspartate
SJS: Stevens-Johnson syndrome
SOD: Superoxide Dismutase
TCC: Trigeminal Cervical Complex
TNC: Trigeminal Nucleus Caudalis
VPA: Valproic Acid

## References

[1] Vécsei L., Lukács M., Tajti J., Fülöp F., Toldi J., Edvinsson L. (2019). The therapeutic impact of new migraine discoveries.. Curr. Med. Chem..

[2] Hoffmann J., Charles A. (2018). Glutamate and its receptors as therapeutic targets for migraine.. Neurotherapeutics.

[3] Chan K., MaassenVanDenBrink A. (2014). Glutamate receptor antagonists in the management of migraine.. Drugs.

[4] Goadsby P.J., Holland P.R. (2019). Pathophysiology of Migraine.. Neurol. Clin..

[5] Gasparini C.F., Smith R.A., Griffiths L.R. (2016). Genetic insights into migraine and glutamate: A protagonist driving the headache.. J. Neurol. Sci..

[6] Lauritzen M., Hansen A.J. (1992). The effect of glutamate receptor blockade on anoxic depolarization and cortical spreading depression.. J. Cereb. Blood Flow Metab..

[7] Ashina M. (2020). Migraine.. N. Engl. J. Med..

[8] Lupi C., Benemei S., Guerzoni S., Pellesi L., Negro A. (2019). Pharmacokinetics and pharmacodynamics of new acute treatments for migraine.. Expert Opin. Drug Metab. Toxicol..

[9] Schytz H.W., Hargreaves R., Ashina M. (2017). Challenges in developing drugs for primary headaches.. Prog. Neurobiol..

[10] Barbanti P., Aurilia C., Egeo G., Fofi L. (2012). Future trends in drugs for migraine prophylaxis.. Neurol. Sci..

[11] Tajti J., Csáti A., Vécsei L. (2014). Novel strategies for the treatment of migraine attacks via the CGRP, serotonin, dopamine, PAC1, and NMDA receptors.. Expert Opin. Drug Metab. Toxicol..

[12] Aurora S.K., Dodick D.W., Turkel C.C., DeGryse R.E., Silberstein S.D., Lipton R.B., Diener H.C., Brin M.F. (2010). PREEMPT 1 Chronic Migraine Study Group. Onabotulinumtoxin A for treatment of chronic migraine: Results from the double-blind; randomized.; placebo-controlled phase of the PREEMPT 1 trial.. Cephalalgia.

[13] Diener H.C., Dodick D.W., Aurora S.K., Turkel C.C., DeGryse R.E., Lipton R.B., Silberstein S.D., Brin M.F. (2010). PREEMPT 2 Chronic Migraine Study Group. Onabotulinumtoxin A for treatment of chronic migraine: Results from the double-blind.; randomized.; placebo-controlled phase of the PREEMPT 2 trial.. Cephalalgia.

[14] Ferrari A., Spaccalopelo L., Pinetti D., Tacchi R., Bertolini A. (2009). Effective prophylactic treatments of migraine lower plasma glutamate levels.. Cephalalgia.

[15] Vieira D.S., Naffah-Mazzacoratti M.G., Zukerman E., Soares C.A.S., Cavalheiro E.A., Peres M.F.P. (2007). Glutamate levels in cerebrospinal fluid and triptans overuse in chronic migraine.. Headache.

[16] Michail Vikelis, Mitsikostas D.D. (2007). The role of glutamate and its receptors in migraine.. CNS Neurol. Disord. Drug Targets.

[17] Ramadan N.M. (2003). The link between glutamate and migraine.. CNS Spectr..

[18] Kew J.N.C., Kemp J.A. (2005). Ionotropic and metabotropic glutamate receptor structure and pharmacology.. Psychopharmacology (Berl.).

[19] Traynelis S.F., Wollmuth L.P., McBain C.J., Menniti F.S., Vance K.M., Ogden K.K., Hansen K.B., Yuan H., Myers S.J., Dingledine R. (2010). Glutamate receptor ion channels: Structure, regulation, and function.. Pharmacol. Rev..

[20] Mazzitelli M., Presto P., Antenucci N., Meltan S., Neugebauer V. (2022). Recent advances in the modulation of pain by the metabotropic glutamate receptors.. Cells.

[21] Reiner A., Levitz J. (2018). Glutamatergic signaling in the central nervous system: Ionotropic and metabotropic receptors in concert.. Neuron.

[22] Burstein R. (2001). Deconstructing migraine headache into peripheral and central sensitization.. Pain.

[23] Burstein R., Cutrer M.F., Yarnitsky D. (2000). The development of cutaneous allodynia during a migraine attack Clinical evidence for the sequential recruitment of spinal and supraspinal nociceptive neurons in migraine.. Brain.

[24] Burstein R., Yarnitsky D., Goor-Aryeh I., Ransil B.J., Bajwa Z.H. (2000). An association between migraine and cutaneous allodynia.. Ann. Neurol..

[25] Park C.G., Chu M.K. (2022). Interictal plasma glutamate levels are elevated in individuals with episodic and chronic migraine.. Sci. Rep..

[26] Baad-Hansen L., Cairns B.E., Ernberg M., Svensson P. (2010). Effect of systemic monosodium glutamate (MSG) on headache and pericranial muscle sensitivity.. Cephalalgia.

[27] Shimada A., Cairns B.E., Vad N., Ulriksen K., Pedersen A.M.L., Svensson P., Baad-Hansen L. (2013). Headache and mechanical sensitization of human pericranial muscles after repeated intake of monosodium glutamate (MSG).. J. Headache Pain.

[28] Liang Y.C., Huang C.C., Hsu K.S. (2005). Characterization of long-term potentiation of primary afferent transmission at trigeminal synapses of juvenile rats: Essential role of subtype 5 metabotropic glutamate receptors.. Pain.

[29] Hoffmann J., Martins-Oliveira M., Supronsinchai W. (2014). The CK1δ T44A mutation affects nociceptive activation of the trigeminocervical complex in an in vivo model of migraine (P1.258).. Neurology.

[30] Ladépêche L., Dupuis J.P., Groc L. (2014). Surface trafficking of NMDA receptors: Gathering from a partner to another.. Semin. Cell Dev. Biol..

[31] Collingridge G.L., Bliss T.V.P. (1995). Memories of NMDA receptors and LTP.. Trends Neurosci..

[32] Rison R.A., Stanton P.K. (1995). Long-term potentiation and N-methyl-d-aspartate receptors: Foundations of memory and neurologic disease?. Neurosci. Biobehav. Rev..

[33] Ahmed H., Haider A., Ametamey S.M. (2020). N -Methyl-D-Aspartate (NMDA) receptor modulators: A patent review (2015-present).. Expert Opin. Ther. Pat..

[34] Parsons C.G., Danysz W., Quack G. (1999). Memantine is a clinically well tolerated N-methyl-d-aspartate (NMDA) receptor antagonist—a review of preclinical data.. Neuropharmacology.

[35] Paoletti P. (2011). Molecular basis of NMDA receptor functional diversity.. Eur. J. Neurosci..

[36] O’Brien M., Cairns B.E. (2016). Monosodium glutamate alters the response properties of rat trigeminovascular neurons through activation of peripheral NMDA receptors.. Neuroscience.

[37] Guerrero-Toro C., Koroleva K., Ermakova E., Ga furov O., Abushik P., Tavi P., Sitdikova G., Giniatullin R. (2022). Testing the role of glutamate NMDA receptors in peripheral trigeminal nociception im plicated in migraine pain.. Int. J. Mol. Sci..

[38] Zhou Q., Wang J., Zhang X., Zeng L., Wang L., Jiang W. (2013). Effect of metabotropic glutamate 5 receptor antagonists on morphine efficacy and tolerance in rats with neuropathic pain.. Eur. J. Pharmacol..

[39] Rogawski M.A. (2008). Common pathophysiologic mechanisms in migraine and epilepsy.. Arch. Neurol..

[40] Latremoliere A., Woolf C.J. (2009). Central sensitization: A generator of pain hypersensitivity by central neural plasticity.. J. Pain.

[41] Tallaksen-Greene S.J., Young A.B., Penney J.B., Beitz A.J. (1992). Excitatory amino acid binding sites in the trigeminal principal sensory and spinal trigeminal nuclei of the rat.. Neurosci. Lett..

[42] Huang T.H., Lai M.C., Chen Y.S., Huang C.W. (2023). The roles of glutamate receptors and their antagonists in status epilepticus, refractory status epilepticus, and super-refractory status epilepticus.. Biomedicines.

[43] Zhang W., Lei M., Wen Q., Zhang D., Qin G., Zhou J., Chen L. (2022). Dopamine receptor D2 regulates GLUA1-containing AMPA receptor trafficking and central sensitization through the PI3K signaling pathway in a male rat model of chronic migraine.. J. Headache Pain.

[44] Leo A., Giovannini G., Russo E., Meletti S. (2018). The role of AMPA receptors and their antagonists in status epilepticus.. Epilepsia.

[45] Guo C., Ma Y.Y. (2021). Calcium permeable-AMPA receptors and excitotoxicity in neurological disorders.. Front. Neural Circuits.

[46] Man H.Y. (2011). GluA2-lacking, calcium-permeable AMPA receptors-inducers of plasticity?.. Curr. Opin. Neurobiol..

[47] Gilron I., Max M.B., Lee G., Booher S.L., Sang C.N., Chappell A.S., Dionne R.A. (2000). Effects of the 2-amino-3-hydroxy-5-methyl-4-isoxazole-proprionic acid/kainate antagonist LY293558 on spontaneous and evoked postoperative pain.. Clin. Pharmacol. Ther..

[48] Chan K.Y., Gupta S., de Vries R., Danser A.H., Villalón C.M., Muñoz-Islas E. (2010). Maassenvanden brink, A. Effects of ionotropic glutamate receptor an tagonists on rat dural artery diameter in an intravital microscopy model.. Br. J. Pharmacol..

[49] Chałupnik P., Szymańska E. (2023). Kainate receptor antagonists: Recent advances and therapeutic perspective.. Int. J. Mol. Sci..

[50] Hansen K.B., Wollmuth L.P., Bowie D., Furukawa H., Menniti F.S., Sobolevsky A.I., Swanson G.T., Swanger S.A., Greger I.H., Nakagawa T., McBain C.J., Jayaraman V., Low C.M., Dell’Acqua M.L., Diamond J.S., Camp C.R., Perszyk R.E., Yuan H., Traynelis S.F. (2021). Structure, function, and pharmacology of glutamate receptor ion channels.. Pharmacol. Rev..

[51] Huettner J.E. (2003). Kainate receptors and synaptic transmission.. Prog. Neurobiol..

[52] Zhou S., Bonasera L., Carlton S.M. (1996). Peripheral administration of NMDA, AMPA or KA results in pain behaviors in rats.. Neuroreport.

[53] Jackson D.L., Graff C.B., Richardson J.D., Hargreaves K.M. (1995). Glutamate participates in the peripheral modulation of thermal hyperalgesia in rats.. Eur. J. Pharmacol..

[54] Bleakman D., Alt A., Nisenbaum E.S. (2006). Glutamate receptors and pain.. Semin. Cell Dev. Biol..

[55] Walker K., Reeve A., Bowes M., Winter J., Woth erspoon G., Davis A., Schmid P., Gasparini F., Kuhn R., Urban L. (2001). mGlu5 receptors and nocicep tive function II. mGlu5 receptors functionally ex pressed on peripheral sensory neurones mediate in flammatory hyperalgesia.. Neuropharmacology.

[56] Walker K., Bowes M., Panesar M., Davis A. (2001). Metabotropic glutamate receptor sub type 5 (mGlu5) and nociceptive function. I. Selective blockade of mGlu5 receptors in models of acute, per sistent and chronic pain.. Neuropharmacology.

[57] Waung M.W., Akerman S., Wakefield M., Keywood C., Goadsby P.J. (2016). Metabotropic glutamate receptor 5: A target for migraine therapy.. Ann. Clin. Transl. Neurol..

[58] Copeland C.S., Neale S.A., Nisenbaum E.S., Salt T.E. (2022). Group II metabotropic glutamate receptor (MGLU 2 and MGLU 3) roles in thalamic processing.. Br. J. Pharmacol..

[59] Bereiter D.A., Bereiter D.F., Hathaway C.B. (1996). The NMDA receptor antagonist MK-801 reduces Fos-like immunoreactivity in central trigeminal neurons and blocks select endocrine and autonomic responses to corneal stimulation in the rat.. Pain.

[60] Classey J.D., Knight Y.E., Goadsby P.J. (2001). The NMDA receptor antagonist MK-801 reduces Fos-like immunoreactivity within the trigeminocervical complex following superior sagittal sinus stimulation in the cat.. Brain Res..

[61] Hattori Y., Watanabe M., Iwabe T., Tanaka E., Nishi M., Aoyama J., Satoda T., Uchida T., Tanne K. (2004). Administration of MK-801 decreases c-Fos expression in the trigeminal sensory nuclear complex but increases it in the midbrain during experimental movement of rat molars.. Brain Res..

[62] Mitsikostas D.D., del Rio M.S., Waeber C., Moskowitz M.A., Cutrer M.F. (1998). The NMDA receptor antagonist MK-801 reduces capsaicin-induced c-fos expression within rat trigeminal nucleus caudalis.. Pain.

[63] Nagy-Grócz G., Zádor F., Dvorácskó S., Bohár Z., Benyhe S., Tömböly C., Párdutz Á., Vécsei L. (2017). Interactions between the kynurenine and the endocannabinoid system with special emphasis on migraine.. Int. J. Mol. Sci..

[64] Vécsei L., Majláth Z., Balog A., Tajti J. (2015). Drug targets of migraine and neuropathy: Treatment of hyperexcitability.. CNS Neurol. Disord. Drug Targets.

[65] Guo S., Vecsei L., Ashina M. (2011). The L-kynurenine signalling pathway in trigeminal pain processing: A potential therapeutic target in migraine?. Cephalalgia.

[66] Al-Karagholi M.A.M., Hansen J.M., Abou-Kassem D., Hansted A.K., Ubhayasekera K., Bergquist J., Vécsei L., Jansen-Olesen I., Ashina M. (2021). Phase 1 study to access safety, tolerability, pharmacokinetics, and pharmacodynamics of kynurenine in healthy volunteers.. Pharmacol. Res. Perspect..

[67] Birch P.J., Grossman C.J., Hayes A.G. (1988). Kynurenic acid antagonises responses to NMDA via an action at the strychnine-insensitive glycine receptor.. Eur. J. Pharmacol..

[68] Fukui S., Schwarcz R., Rapoport S.I., Takada Y., Smith Q.R. (1991). Blood-brain barrier transport of kynurenines: Implications for brain synthesis and metabolism.. J. Neurochem..

[69] Colpo G.D., Venna V.R., McCullough L.D., Teixeira A.L. (2019). Systematic review on the involvement of the kynurenine pathway in stroke: Pre-clinical and clinical evidence.. Front. Neurol..

[70] Vécsei L., Beal M.F. (1991). Comparative behavioral and pharmacological studies with centrally administered kynurenine and kynurenic acid in rats.. Eur. J. Pharmacol..

[71] Gomez-Mancilla B., Brand R., Jurgens T.P., Gobel H., Sommer C., Straube A., Evers S., Sommer M., Campos V., Kalkman H.O., Hariry S., Pezous N., Johns D., Diener H-C. (2014). Randomized, multicenter trial to assess the efficacy, safety and tolerability of a single dose of a novel AMPA receptor antagonist BGG492 for the treatment of acute migraine attacks.. Cephalalgia.

[72] Ramadan N.M. (2014). Glutamate and migraine: From Ikeda to the 21st century.. Cephalalgia.

[73] Sahara Y., Noro N., Iida Y., Soma K., Nakamura Y. (1997). Glutamate receptor subunits GluR5 and KA-2 are coexpressed in rat trigeminal ganglion neurons.. J. Neurosci..

[74] Johnson K.W., Nisenbaum E.S., Johnson M.P., Dieckman D.K., Clemens-Smith A., Siuda E.R. (2008). In novative drug development for headache disorders: glutamate.. Frontiers in Headache Research Series.

[75] Andreou A.P., Holland P.R., Lasalandra M.P., Goadsby P.J. (2015). Modulation of nociceptive dural input to the trigeminocervical complex through GluK1 kainate receptors.. Pain.

[76] Andreou A.P., Holland P.R., Goadsby P.J. (2009). Activation of iGluR5 kainate receptors inhibits neurogenic dural vasodilatation in an animal model of trigeminovascular activation.. Br. J. Pharmacol..

[77] Sang C.N., Ramadan N.M., Wallihan R.G., Chappell A.S., Freitag F.G., Smith T.R., Silberstein S.D., Johnson K.W., Phebus L.A., Bleakman D., Ornstein P.L., Arnold B., Tepper S.J., Vandenhende F. (2004). LY293558, a novel AMPA/GluR5 antagonist, is efficacious and well-tolerated in acute migraine.. Cephalalgia.

[78] Witkin J.M., Pandey K.P., Smith J.L. (2022). Clinical investigations of compounds targeting metabotropic glutamate receptors.. Pharmacol. Biochem. Behav..

[79] Marin J.C.A., Goadsby P.J. (2010). Glutamatergic fine tuning with ADX-10059: A novel therapeutic approach for migraine?. Expert Opin. Investig. Drugs.

[80] Terzioglu B., Oguz E., Acet G. (2020). Effect of valproic acid on oxidative stress parameters of glutamate induced excitotoxicity in SH SY5Y cells.. Exp. Ther. Med..

[81] Chaudhary S., Parvez S. (2012). An in vitro approach to assess the neurotoxicity of valproic acid-induced oxidative stress in cerebellum and cerebral cortex of young rats.. Neuroscience.

[82] Sun Z.W., Zhang L., Zhu S.J., Chen W.C., Mei B. (2010). Excitotoxicity effects of glutamate on human neuroblastoma SH-SY5Y cells via oxidative damage.. Neurosci. Bull..

[83] Lee J.Y., Maeng S., Kang S.R., Choi H.Y., Oh T.H., Ju B.G., Yune T.Y. (2014). Valproic acid protects motor neuron death by inhibiting oxidative stress and endoplasmic reticulum stress-mediated cytochrome C release after spinal cord injury.. J. Neurotrauma.

[84] Diener H.C., Tfelt-Hansen P., Dahlöf C., Láinez M.J., Sandrini G., Wang S.J., Neto W., Vijapurkar U., Doyle A., Jacobs D. (2004). Topiramate in migraine prophylaxis--results from a placebo-controlled trial with propranolol as an active control.. J. Neurol..

[85] Zhang X., Velumian A.A., Jones O.T., Carlen P.L. (2000). Modulation of high-voltage-activated calcium channels in dentate granule cells by topiramate.. Epilepsia.

[86] Taverna S., Sancini G., Mantegazza M., Frances chetti S., Avanzini G. (1999). Inhibition of transient and persistent Na+ current fractions by the new anti covulsant topiramate.. J. Pharmacol. Exp. Ther..

[87] White H.S., Brown S.D., Woodhead J.H., Skeen G.A., Wolf H.H. (1997). Topiramate enhances GABA-mediated chloride flux and GABA-evoked chloride currents in murine brain neurons and increases seizure threshold.. Epilepsy Res..

[88] Kaminski R.M., Banerjee M., Rogawski M.A. (2004). Topiramate selectively protects against seizures induced by ATPA, a GluR5 kainate receptor agonist.. Neuropharmacology.

[89] Sprenger T., Viana M., Tassorelli C. (2018). Current prophylactic medications for migraine and their potential mechanisms of action.. Neurotherapeutics.

[90] Steiner T.J., Findley L.J., Yuen A.W.C. (1997). Lamotrigine versus placebo in the prophylaxis of migraine with and without aura.. Cephalalgia.

[91] Lampl C., Buzath A., Klinger D., Neumann K. (1999). Lamotrigine in the prophylactic treatment of migraine aura-a pilot study.. Cephalalgia.

[92] D’Andrea G., Granella F., Cadaldini M., Manzoni G.C. (1999). Effectiveness of lamotrigine in the prophylaxis of migraine with aura: an open pilot study.. Cephalalgia.

[93] Hansen J.M., Charles A. (2019). Differences in treatment response between migraine with aura and migraine without aura: Lessons from clinical practice and RCTs.. J. Headache Pain.

[94] Iacobucci G.J., Visnjevac O., Pourafkari L., Nader N.D. (2017). Ketamine: An update on cellular and subcellular mechanisms with implications for clinical practice.. Pain Physician.

[95] Rueda Carrillo L., Garcia K.A., Yalcin N., Shah M. (2022). Ketamine and its emergence in the field of neurology.. Cureus.

[96] Etchison A., Bos L., Ray M., McAllister K., Mohammed M., Park B., Phan A., Heitz C. (2018). Low-dose ketamine does not improve migraine in the emergency department: A randomized placebo-controlled trial.. West. J. Emerg. Med..

[97] Nicolodi M., Sicuteri F. (1995). Exploration of NMDA receptors in migraine: Therapeutic and theoretic implications.. Int. J. Clin. Pharmacol. Res..

[98] Wohleb E.S., Gerhard D., Thomas A., Duman R.S. (2017). Molecular and cellular mechanisms of rapid-acting antidepressants ketamine and scopolamine.. Curr. Neuropharmacol..

[99] Rammes G., Rupprecht R., Ferrari U., Zieglgänsberger W., Parsons C.G. (2001). The N-methyl-d-aspartate receptor channel blockers memantine, MRZ 2/579 and other amino-alkyl-cyclohexanes antagonise 5-HT3 receptor currents in cultured HEK-293 and N1E-115 cell systems in a non-competitive manner.. Neurosci. Lett..

[100] Kabir M.T., Sufian M.A., Uddin M.S., Begum M.M., Akhter S., Islam A., Mathew B., Islam M.S., Amran M.S., Md Ashraf G. (2019). NMDA receptor antagonists: Repositioning of memantine as a multitargeting agent for Alzheimer’s therapy.. Curr. Pharm. Des..

[101] Mistry V.M., Morizio P.L., Pepin M.J., Bryan W.E., Brown J.N. (2021). Role of memantine in the prophylactic treatment of episodic migraine: A systematic review.. Headache.

[102] Shanmugam S., Karunaikadal K., Varadarajan S., Krishnan M. (2019). Memantine ameliorates migraine headache.. Ann. Indian Acad. Neurol..

[103] Bigal M., Rapoport A., Sheftell F., Tepper D., Tepper S. (2008). Memantine in the preventive treatment of refractory migraine.. Headache.

[104] Bentivegna E., Luciani M., Ferrari V., Galastri S., Baldari F., Scarso F., Lamberti P.A., Martelletti P. (2022). Recently approved and emerging drug options for migraine prophylaxis.. Expert Opin. Pharmacother..

[105] Zhou T., Tang Y., Zhu H. (2022). Effectiveness and safety of memantine for headache: A meta-analysis of randomized controlled studies.. Clin. Neuropharmacol..

[106] Domitrz I., Cegielska J. (2022). Magnesium as an important factor in the pathogenesis and treatment of migraine—from theory to practice.. Nutrients.

[107] Shin H.J., Na H.S., Do S.H. (2020). Magnesium and Pain.. Nutrients.

[108] Ahmed F., Mohammed A. (2019). Magnesium: The forgotten electrolyte—a review on hypomagnesemia.. Med. Sci. (Basel).

[109] Arpaci D., Tocoglu A.G., Ergenc H., Korkmaz S., Ucar A., Tamer A. (2015). Associations of serum Magnesium levels with diabetes mellitus and diabetic complications.. Hippokratia.

[110] Hanada T., Ido K., Kosasa T. (2014). Effect of perampanel, a novel AMPA antagonist, on benzodiazepine-resistant status epilepticus in a lithium-pilocarpine rat model.. Pharmacol. Res. Perspect..

[111] Fernandes M., Dono F., Dainese F., Renna R., Consoli S., Gaspari C., Izzi F., Pagliuca M., Placidi F., Biagio Mercuri N., Liguori C. (2021). Perampanel may represent an effective treatment for the preven tion of migraine comorbid with epilepsy.. Epilepsy Behav..

[112] Lim S.N., Wu T., Tseng W.E.J., Chiang H.I., Cheng M.Y., Lin W.R., Lin C.N. (2021). Efficacy and safety of perampanel in refractory and super-refractory status epilepticus: cohort study of 81 patients and literature review.. J. Neurol..

[113] Rugg-Gunn F. (2014). Adverse effects and safety profile of perampanel: A review of pooled data.. Epilepsia.

